# Necroptosis has a crucial role in the development of chronic periodontitis

**DOI:** 10.1016/j.jobcr.2023.05.010

**Published:** 2023-05-23

**Authors:** Salehe Akhondian, Kazem Fatemi, Niloofar Ebrahim Zadeh, Seyed Abdolrahim Rezaee, Sahar Bayat, Zahra Shooshtari, Farnaz Mohajertehran

**Affiliations:** a Faculty of Dentistry, Mashhad University of Medical Science, Mashhad, Iran; b Oral and Maxillofacial Disease Research Center, Mashhad University of Medical Sciences, Mashhad, Iran; c Faculty of Dentistry, Zahedan University of Medical Science, Zahedan, Iran; d Immunology Research Center, Inflammation and Inflammatory Diseases Division, Mashhad University of Medical Sciences, Mashhad, Iran; e Dental Research Center, Mashhad Dental School, Mashhad University of Medical Sciences, Mashhad, Iran

**Keywords:** Chronic periodontitis, Receptor-interacting protein kinase-1, Receptor-interacting serine-threonine kinase-3 protein, Cell death, Necroptosis

## Abstract

**Background and aim:**

Periodontitis is a non-communicable chronic inflammatory disease that affects the entire periodontium and its severe types cause irreparable destruction. The purpose of this study was to determine the type of cell death in chronic periodontitis (CP) with the expression of receptor-interacting protein kinase (RIPK) type1 and RIPK3 genes.

**Materials and methods:**

This cross-sectional study was carried out from September 2019 to 2020. The samples (38 participants) were divided into two groups: 20 recently diagnosed CP patients and 18 healthy individuals. Participants' data was collected in the periodontology Department, Dental school, Mashhad University of Medical Sciences and sent to the Immunology Lab for assessment of RIPK1 and RIPK3 expressions using quantitative real time-PCR.

**Results:**

The study sample consisted of 30 females (78.9%) and 8 males (21.1%) with a mean age of 34 ± 5 years. The expression of the genes of interest in CPs exhibited an opposite pattern. Although, RIPK3 gene expression was significantly greater in CP patients compared to the control group (P = 0.024), the expression of RIPK1 decreased (p < 0.001). Moreover, no significant correlation was observed between age and gender with these molecules in CPs.

**Conclusion:**

The RIPK3 selectively contributes to necroptosis, therefore, it seems that RIPK3-mediated necroptosis is involved in chronic periodontitis. RIPK1 also participates in necroptosis, but mostly in apoptosis. Therefore, necroptosis as an unprogrammed inflammatory cell death induced by pathogenic damages seems to be another mechanism complicated in periodontitis and could be used as a novel target for CP therapy.

## 1 Introduction

Periodontal disease has long been considered to be a silent pandemic with a complex multifactorial etiology and evidence-based assertions of immune-mediated pathogenesis.^1^ According to recent studies, the incidence rate of periodontal disease has risen to 57.3%, indicating a prominent increasing trend.^2^ Thereby, effective periodontal therapy remains a long-term and challenging endeavor.^2^ Gingivitis and periodontitis are the two main manifestations of periodontal disease.^3^ Periodontitis, a widespread infectious oral disease, yet cannot be fully prevented.^4^ This disorder is associated with an overproduction of inflammatory cytokines, and irreversible loss of connective tissue and alveolar bone support, degradation of the periodontal ligament, and attachment breakdown.^4^^,^^5^ Arguably, COVID-19 complications were shown to be significantly more common in patients with moderate-to-severe periodontitis.^6^

Chronic periodontitis (CP)is the most prevalent form of periodontitis and the leading cause of tooth loss in two‐thirds (68%) of individuals aged 65 years; however, it can also affect children and adolescents.^5^^,^^7^ This type of periodontitis is reported to be the sixth-most common illness, affecting over 700 million people worldwide.^8^ It can also jeopardize one's Oral Health-Related Quality of Life (OHRQoL), diet, speech, social relationships, and psychological well-being, and is associated with several systemic diseases.^8^ Dental plaque is a key cause of chronic periodontitis, as it damages periodontal tissue and triggers an inflammatory and immunological response.^9^

The processes responsible for the progression of periodontal disease remain uncertain^10^. Cell death, traditionally thought to be the consequence of inflammation, has now been linked to the etiology of inflammatory disorders.^11^ Programmed cell death (PCD), unlike necrosis, is an active cell death regulated by a series of gene expression processes and can be mainly classified into apoptosis, autophagy, necroptosis, and pyroptosis.^4^

Apoptosis is a critical regulator of the host immune system that is affected by cytokines, bacterial and viral infections, immune cells themselves, and the extracellular matrix.^10^ Unlike apoptosis, necrosis is an uncontrolled process characterized by a rapid loss of membrane integrity, release of extracellular contents, severe inflammation, and immune system activation.^12^

Necroptosis is a recently discovered mechanism of regulated necrosis that involves the proteins receptor-interacting protein serine-threonine kinases-3 (RIPK3) and mixed lineage kinase domain-like protein (MLKL).^13^ In contrast to apoptosis, which is immune-suppressive, necroptosis is a highly inflammatory process.^14^ Periodontitis has yet to be definitively proven to be caused by necroptosis.^4^^,^^14^

Gram-negative bacteria, including Porphyromonas gingivalis and B. firsitus, are the most common causes of periodontal infection.^15^^,^^16^ The presence of gram-negative bacteria and lipopolysaccharide (LPS) by inducing Tumor necrosis factor-alpha (TNF-α), enhances the risk of necroptosis in periodontitis.^16^ This might be one of the probable roles of necroptosis in CP.

Several debates were noted among researchers. According to prior studies, many apoptotic agents have a high sensitivity and positive prediction capability for periodontal disease.^10^ Several investigations have indicated that inflamed periodontal tissue has a lower apoptosis rate.^17^^,^^18^ Recent trials indicated that RIPK3 and MLKL-mediated necroptotic cell death played a role in the development of periodontitis.^19^

Considering the lack of adequate research on this subject in Iran and the numerous controversies listed above, the authors aimed to determine the type of cell death in chronic periodontitis with the expression of RIPK1 and RIPK3 genes.

## 2 Methods and materials

### 2.1 Study design and sample collection

This cross-sectional study was carried out from September 2019 to 2020 on 20 patients with chronic periodontitis as the case group and 18 healthy subjects in terms of periodontal condition as the control group among those who referred to the Periodontology Department of Mashhad Dental School, Mashhad University of Medical Sciences (MUMS), Mashhad, Iran Laboratory analyses were performed in the immunology department Written consent was submitted from the participants prior to enrollment. The study's protocol was approved by the Ethical Committee for Biomedical research, MUMS (IR.MUMS.REC.1398.222).

This research did not involve any sort of intervention. Inclusion criteria were at least 18 years of age, a recent diagnosis of chronic periodontitis without any previous treatment and having at least 10 teeth in the mouth.^20^ Participants who had conditions such as metabolic diseases, allergies, smoking, pregnancy, breastfeeding, use of systemic or topical antibiotics in the last 3 months, consuming drugs such as contraceptives, immunosuppressants, and those affecting periodontal conditions, were excluded from the study. Therefore, patients with these interfering factors were excluded from the study. Participants were standardized in terms of age and sex in both periodontitis and healthy groups.

PI (plaque index), BOP (bleeding on probing), and CAL (clinical attachment level) were evaluated by using dental probes. The diagnosis of chronic periodontitis was established based on having a minimum germ-free depth and CAL of more than 4 mm and having BOP. CAL by measuring the distance between Cemento-Enamel Junction (CEJ) to a depth of the gingival sulcus was determined for each tooth. Silness and loe method.^21^ was used to detect the presence of dental plaque. A tissue sample was obtained from each and every individual. In the case group, the tissue sample was taken from the area affected by chronic periodontitis. Whereas in the control group, a healthy area of tissue excised during third molar surgery or esthetic crown lengthening surgery was used. In order to standardize the samples, all biopsies were taken from the facial papilla, and also sampling was not performed around teeth with endo-periosteal lesions, occlusal disharmony, pericoronitis, and the pathological lesions or third molars.

The samples were immediately converted to cryotubes containing RNA later solution (Qiagen, Germany) and sent to the Immunology laboratory, Mashhad School of Medicine, MUMS, and then stored at-70 °C for RNA extraction and cDNA synthesis.

### 2.2 RNA extraction

**Fig. 1:**
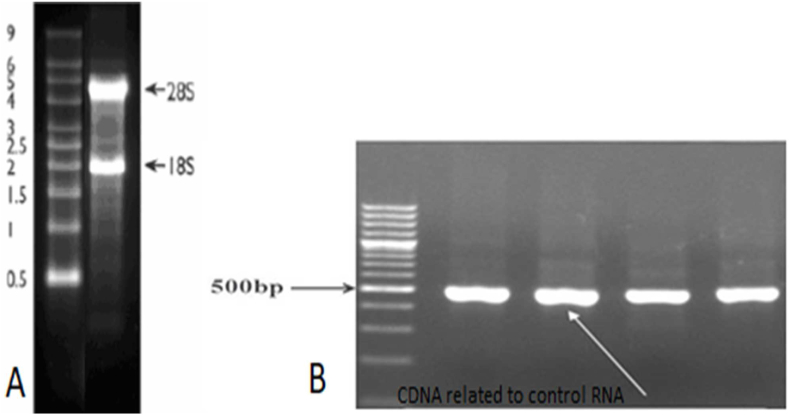
A) For quality control and integrity test of RNA extracted samples, electrophoresis on 2% agarose gel done and S18 and S28 sharp bands was observed *B) B) For quality control of cDNA synthesis, PCR test done with GAPDH-Control-RNA in Thermo Scientific RevertAid First Strand cDNA Synthesis Kit for 4 samples*

Fresh tissue was prepared and lysed to examine the expression of RIPK1 and RIPK3 genes. For this purpose, total RNA was extracted from each sample according to the Total RNA Extraction Kit instructions (Pars Tous Company, Mashhad, Iran) then total RNA was run on 2% agarose gel to see 28 S and 18 S ribosomal RNA (rRNA) bands (Fig. 1).

An absorbance ratio of 260/280 nm was used to assess the purity of extracted RNA using a NanoDrop 2000 spectrophotometer (NanoDrop Technologies, Wilmington, Thermo, USA). The 260/280 nm ratio of ∼2.0 is considered for pure RNA.

### 2.3 cDNA synthesis

For each sample, 480 ng of total RNA was used for cDNA synthesis according to the manufacturer instructions of the Thermo Scientific Revert Aid First Strand cDNA Synthesis Kit (Thermo Scientific, USA). The cDNA synthesis was performed in a total volume of 20 μl according to the manufacturer's instructions, including 10 μl of 2X reaction buffer, 2 μl of 20X enzyme, 8 μl of RNA, using ABI Thermocycler (One Step, USA). The temperature cycle protocol included pre-incubation at 25 °C for 10 min, reverse transcription (RT) at 50 °C for 60 min, RT inactivation at 80 °C for 5 min, and holding at 12.

### 2.4 Quantitative real-time PCR (qPCR)

QPCR was used for gene expression analysis. The relative expression of RIPK1 and RIPK3 genes compared to the beta-2 microglobulin (β2M) reference gene was performed by using the Eva green Master mix kit (Qiagen, Germany). The Primers used in this study were designed with Beacon Designer (PREMIER Biosoft, USA) and checked with Nucleotide and PCR Blast (NIH.NLM. Blast, USA), and they were sent for synthesis (Table 1). The validity of the primers was confirmed with conventional PCR.

**Table 1:** Sequence of primers.

Target gene	Sequence (5'→3′)	length	Product size	RefSeq
RIPK1	F: 5′-GGGAAGGTGTCTCTGTGTTTC-3′	21	91 bp	NM_001354930.2
	R: 5′-CCTCGTTGTGCTCAATGCAG-3′	20		
RIPK3	F: 5′-ATGTCGTGCGTCAAGTTATGG-3′	21	136 bp	NM_006871.4
	R: 5′-CGTAGCCCCACTTCCTATGTTG-3′	21		
Beta2 microglobulin	F: 5′-TTGTCTTTCAGCAAGGACTGG-3′	21	127 bp	NM_004048.4
	R: 5′-CCACTTAACTATCTTGGGCTGTG-3′	23		

Quantities RT-PCR was performed on the cDNA samples using relative two standard curves methods as previously, described.^21^ A Q 6000 Machine (Qiagen, Germany) was used for qRT-PCR and the results were analyzed by Rotor Gene 6000 software. The concentration of each primer for PCR reaction was between 0.5 μM and 1 μM.

### 2.5 Statistical analysis

At the fault level of type I 5% and the error level of type II 20%, according to shi et al.^18^ study and regarding the acceptable difference of 0.25 for the mean of cell death between groups and using the two-mean comparison formula, a minimum of 15 subjects per group, was required. Which increased to 20 patients in each group for more confidence and a total of 40 participants were included in the study. The collected data on the forms were encoded and imported in to SPSS software (version 11.5 SPSS Inc., Chicago, IL, USA). Data description was performed using appropriate statistical tables and diagrams to express measures of central tendency and dispersion indices and frequency distribution. The Mann-Whitney U statistics was used for data analysis. P-value less than 0.05 was considered statistically significant.

## 3 Results

### 3.1 The demographic data of the subjects

The participants in this study were 40 adults, 20 of whom had chronic periodontitis and 20 of whom were healthy. Due to sample destruction,2 and 3 samples of the control group, respectively, during the RIPK1 and RIPK3 tests were excluded from the study. Thus, RIPK1 and RIPK3 evaluations were performed on 18 and 17 samples in the control group, correspondingly. Among the 38 consecutive patients, there were 30 females (78.9%) and 8 males (21.1%) with a mean age of 34 ± 5 years (25–44). Moreover, the age range in the two groups is the same and equal to 17 years.

Although females comprised 78.4% of the subjects, there was no significant difference in gender distribution between the two groups (Table 2). Furthermore, there was no discernible variation in mean age among groups (Table 2).

**Table 2:** Demographic information of study participants.

Variable		Chronic Periodontitis group (n = 20)	Healthy control group (n = 18)	P-value
**Age**	Average	33.3 (25–42)	35.7 (27–44)	
	Standard deviation	4.7	5.1	0.132*
**Gender**	Female	16 (80%)	14 (77.8%)	
	Male	4 (20%)	4 (22.2%)	1.000**

*P value calculated by independent T-test.

**P value calculated by Fishers exact test.

P < 0.05 was considered significant.

### 3.2 The gene expression data

The Kolmogorov-Smirnov test ascertained that the data from both the RIPK1 and RIPK3 genes were not normally distributed.

**Fig. 2:**
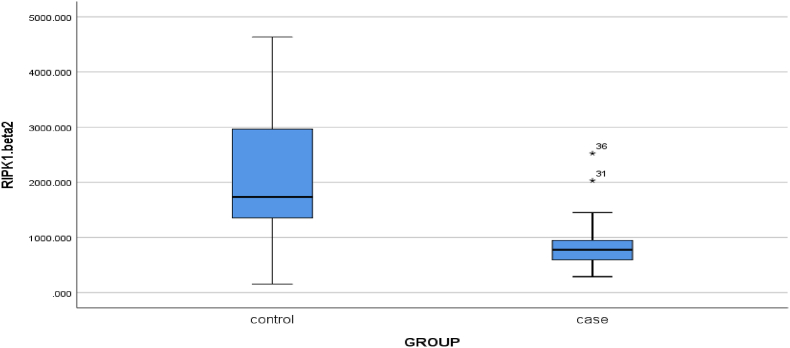
Mean expression of RIPK1 gene in two control groups and patients with chronic periodontitis (P-value<0.001).

**Fig. 3:**
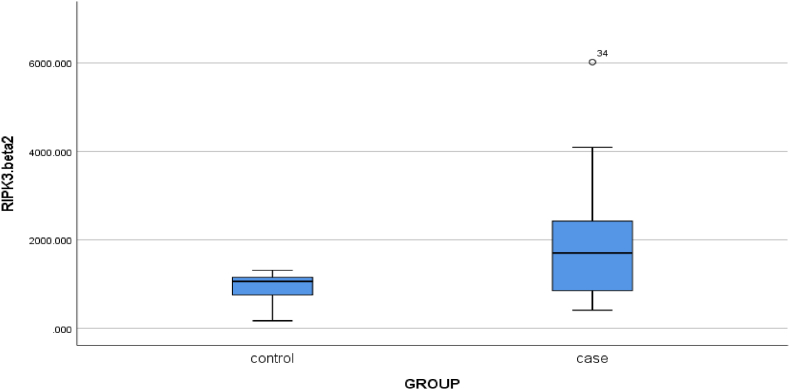
Mean expression of RIPK3 gene in two control groups and patients with chronic periodontitis (P-value = 0.024).

The results illustrate that RIPK1 expression was significantly higher in the control group compared to CP patients, according to the Mann-Whitney *U* test (p < 0.001) (Fig. 2). Furthermore, the same test on RIPK3 expression in patients with chronic periodontitis and the control group, revealed that there was a significant difference between these two groups since the expression of RIPK3 was significantly greater in CP patients compared to the control group (p = 0.024) (Fig. 3).

There were not any significant correlations between the demographic varibales and gene expression data.

### 3.3 Clinical data of the subjects

In the CP group, the mean PI of the patients was evaluated as 65–70%. BOP was evaluated positive in all CP patients with an average of 63–75%. The lowest and highest CAL values in this group were 3.5 and 9 mm, respectively, and the average value was 5.6.

## 4 Discussion

Despite extensive research on the pathogenesis of chronic periodontitis (CP), the type of main cell death associated with the disease still remains unclear, particularly in the presence of a well-known pathogen in this disease, P. gingivalis.^22^

**Fig. 4:**
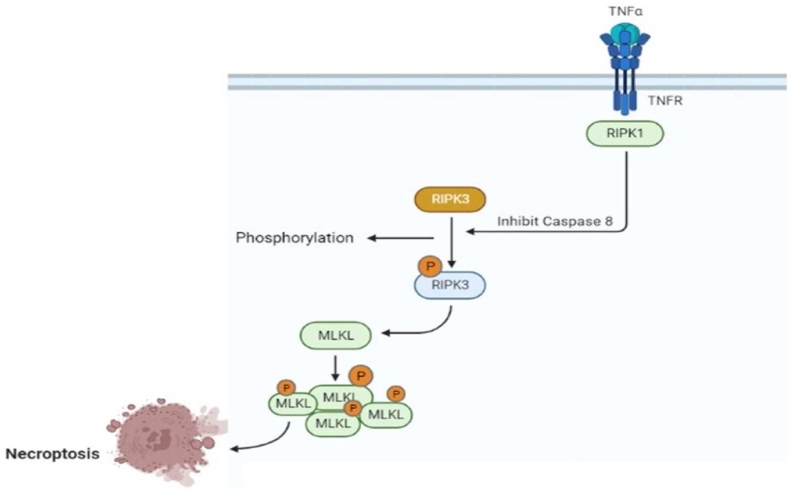
It is clear that TNFR-mediated necroptosis requires both RIP3 and RIP1 kinase activities. However, the formation of a pronecroptotic protein complex, which is independent of RIP1 is also been reported. Thus, the core components of the necroptotic pathway are RIPK3 and MLKL, whereas RIPK1 requirement can be bypassed.

Necroptosis is defined as an un-programmed cell death caused by inflammatory reactions induced by pathogenic damage. Necroptosis has long been an area of clinical research. Therefore, necroptosis is mainly investigated through the activity of necroptotic signaling factors, RIPK1-RIPK3-MLKL, in the cell damages, in our case periodontitis (Fig. 4).

Due to the fact that periodontitis is one of the predisposing factors for systemic diseases, regulating necroptosis created by RIPK1-RIPK3-MLKL is important for timely diagnosis and effective treatment of this disease.^15^

The current study revealed that the expression of receptor-interacting protein serine-threonine kinases −1 (RIPK1) gene in the group with chronic periodontitis was significantly lower than the control group. On the other hand, the expression of the selectively pivotal signaling factor, RIPK3, for inducing necroptosis, was significantly higher in chronic periodontitis than the control group. On the other hand, in the present study, no significant implication of gender or age was found in the occurrence of chronic periodontitis. Furthermore, there were not any correlations between RIPKs expressions and age or gender.

Some studies suggest that periodontitis is more likely to develop in an aging population, due to the global shift toward increased tooth retention in middle aged and older adults.^23^ However, the current study revealed no significant association between age and periodontitis.

Some older research found that male patients experienced greater degrees of periodontal damage compared to females.^24^ The reasons for these sex disparities are unknown, however, they are assumed to be related to males' lack of dental hygiene.^24^ The current study did not reveal any significant relationship between gender and CP.

There are two theories over the type of cell death that causes chronic periodontitis. Apoptotic pathways have been identified in several studies to affect gingival tissue in patients with periodontitis.^25^ It has also been stated that women have the highest incidence of gingival apoptosis in areas with periodontal disease.^26^ In contrast, this study and other recent findings demonstrate that necroptosis is related to the pathogenesis of periodontitis.^18^^,^^27^

In contrast to the present study, Shi et al.,^18^ reported high levels of RIPK1 in gingival tissue collected from patients with untreated chronic periodontitis. An *in vitro* study also found that the expression of RIPK1 in periodontal ligament cells was higher in cases with periodontitis than the control group.^27^ However, our study and some other authors demonstrated, unaffected results or significantly high expression rates of RIPK-1 in the control group. This discrepancy may be due to the different methodology of the conducted studies. For example, the *in vitro* nature of the study by Yan et al.,^27^ or, collecting gingival tissue and healthy sites from the same subjects in Shi et al.^18^ study, might be responsible for these differences. However, in our study, the collected samples were obtained from patients and healthy controls who underwent a routine minor surgery.

As a result of these contradicting findings, it appears that RIPK3 as a selective factor plays more roles in necroptosis of gingival tissue than RIPK1, which also contributed to apoptosis. Therefore, periodontal disease can be described as a RIPK-1 independent factor to gingival damages.^18^

In the current study, the mean expression rate of the RIPK3 gene in the chronic periodontitis group was considerably higher than the control group. This indicated that the periodontal ligament cells have been under the pressure of unprogrammed necroptosis cell death, as the inflammation in the gingival tissue is undeniable. Other researchers have also established similar findings.^27^

Due to the effective role of IL-1B in the occurrence of necroptosis, nonsteroidal anti-inflammatory drugs (NSAIDs) might play a key role in the regulation of necroptosis. Therefore, the attempts for finding appropriate NSAID medications for controlling inflammatory reactions in periodontitis should be brought to the attention of the researchers in this field, as it is also an urgent need for treating the severe oral reactions caused by COVID-19.^19^

One of the limitations of this study was the relatively small sample size. The novelty of this topic may serve as the foundation for future research. Furthermore, because numerous interfering factors decreased the accuracy of the results, these elements were eliminated in this study, and the validity of the data was enhanced. Therefore, some patients were excluded from the study, because the sample size decreased over the course of a year.

## 5 Conclusion

In conclusion, the strong gene expression of RIPK3 in gingival lesions revealed that necroptosis is substantially associated with chronic periodontitis. It is well known that the RIPK3 factor selectively contributes to the necroptosis process,therefore, it seems that RIPK3-mediated necroptosis is associated with chronic periodontitis. RIPK1, not only may participate in necroptosis but is most effective in inducing apoptosis. Therefore, necroptosis seems to be another mechanism involved in periodontitis and could be used as a novel target for CP therapy. These findings support a targeted effort to establish a diagnostic strategy for chronic periodontitis and effective treatment options. Lack of research on the subject of this study, and particularly the investigation of these two genes in chronic periodontitis, may serve as a framework for future research in this area.

## Ethics approval and consent to participate

This study was approved by the Ethics Committee for Biomedical Research, MUMS, (IR.MUMS.REC.1398.222). The authors certify that all data collected during the study are as stated in the manuscript, and no data from the study has been or will be published separately elsewhere.
